# Palliative care for patients with heart failure and family caregivers in rural Appalachia: a randomized controlled trial

**DOI:** 10.1186/s12904-024-01531-2

**Published:** 2024-08-03

**Authors:** Ubolrat Piamjariyakul, Angel Smothers, Kesheng Wang, Saima Shafique, Sijin Wen, Trisha Petitte, Stephanie Young, George Sokos, Carol E. Smith

**Affiliations:** 1 West Virginia University School of Nursing, Morgantown, USA; 2 Department of Biostatistics School of Public Health, West Virginia University, Morgantown, USA; 3 West Virginia University Heart and Vascular Institute, J.W. Ruby Memorial Hospital, Morgantown, USA; 4 School of Nursing, School of Preventive Medicine, University of Kansas Medical Center, Kansas City, USA

**Keywords:** Heart failure, End-of-life, Palliative care, Randomized controlled trial, Appalachia, Family caregivers

## Abstract

**Background:**

Heart failure (HF) is a debilitating disease with worsening symptoms and family caregiving burden. HF affects more than 8 million Americans. West Virginia has the highest HF death rate in the U.S. and limited healthcare services. This study tested whether the family HF palliative and end-of-life care intervention (FamPALcare) improved patient and caregiver outcomes at 3- and 6-month study endpoints.

**Methods:**

This study used a randomized controlled trial design. Patients with HF and their caregivers were randomly assigned together to the intervention (*n* = 21) or control (*n* = 18) group. The intervention included five telephone coaching sessions on the HF home, palliative, and end-of-life care. The outcome data collected at baseline and at 3 and 6 months were from the patients’ (a) HF-related health status and depression/anxiety scale scores; and from caregivers’ (b) caregiving burden and depression/anxiety scale scores; and (c) anonymous ratings on the 11-item FamPALcare helpfulness scale, completed by the intervention participants.

**Results:**

The mean age of the patients was 65.66 (SD = 13.72) years, and 67% were White males. The mean age of the caregivers was 62.05 (SD = 13.14) years, and 77% were White females. Compared to the controls, patients in the intervention group had significantly greater scores for HF-related health status (*p* < .05) and lower depression/anxiety scores at 6 months, the study endpoint. The family caregivers in the intervention group had significantly lower scores on caregiving burden (*p* < .05) and depression/anxiety (*p* < .01) at 3 months. The mean helpfulness rating was M = 4.46 out of 5 (SD = 0.49).

**Conclusions:**

The FamPALcare intervention was found to be effective at improving patient HF-related health status and reducing caregiver burden and improving both patient and caregiver depression and anxiety scores. The FamPALcare HF intervention was found feasible and consistently delivered (fidelity). The FamPALcare intervention’s cost-effectiveness and helpfulness ratings information will be used to plan for subsequent clinical trials.

**Trial registration:**

ClinicalTrials.gov NCT04153890, Registered on 4 November 2019, https://clinicaltrials.gov/ct2/show/NCT04153890.

## Background

Heart failure (HF) affects more than 8 million Americans and is increasing at a rate of 46%, with an increase in costs of up to $70 billion annually [1]. Severe symptoms of HF persist despite medical, surgical, or HF device therapy [2]. HF is a debilitating and life-limiting disease that requires extensive home family caregiving assistance and difficult decisions regarding treatment options for patients who are experiencing progressive decline [3, 4]. Studies verify family caregivers contributions to HF home care. Worsening HF has numerous negative impacts on family caregiver health outcomes, resulting in caregiving burden [3, 5]. In turn, the caregiving burden can have a negative impact on caregiver physical and mental health status [5]. Family caregivers managing patient HF reported unmet needs, [6] lack of communication with health care professionals, [7] and little if any preparedness for home end-of-life and palliative care (EOLPC) [8].

Patients and their families are unprepared for the challenges of this deteriorating condition and for home caregiving burdens and have fears of suffering a painful death [9, 10]. Our nurse-led family home EOLPC intervention (FamPALcare) addresses these challenges [11]. Palliative care includes supportive and comfort care to relieve patient suffering and pain/discomfort [12]. HF palliative care also includes treatments specific for the distressing, commonly repeating physical and psychological symptoms of HF (e.g., breathlessness, fatigue, edema, and depression/anxiety). HF palliative care includes discussions for determining the HF care options according to the families’ end-of-life (EOL) care preferences [13].

Further, patients with HF who live in rural areas have higher mortality rates than those who live in urban areas [14]. Specifically, in West Virginia (WV), where HF death rates are the highest in the country, residents have limited access to healthcare and experience social service inequities [15, 16]. The rural mountainous terrain and distance from HF specialists and local healthcare services increase the family caregivers’ need for more useful home care information [17]. Home palliative care interventions can assist families in managing advancing HF symptoms and end-of-life (EOL) care needs at home [18].

Considering the burden of families managing HF, the increasing prevalence of palliative care needs [19], and the lack of palliative care providers [20], home HF palliative care is understudied with little guidance or information to support caregivers [21, 22]. Thus, family caregivers need to be prepared for complex home care and palliative care specific to HF with considerations for families in rural settings with limited services [23]. Effective palliative care should incorporate patient cultural values and preferences about their disease state and medical treatment [24]. Preparing caregivers for providing HF home care can reduce patient and caregiver anxiety and help them discuss their end-of-life preferences [25].

### Purposes

The purpose of this study was to test whether a home palliative care intervention (FamPALcare) would improve family caregiver and patient HF-related health status and their depression/anxiety scores at 3- and 6-month endpoints. Another purpose was to verify the feasibility, fidelity, helpfulness and costs of FamPALcare remote telephone intervention delivery.

### Conceptual model

We utilized the empirically established coaching model for HF home care as a framework to develop this study’s coaching intervention components [26]. Coaching included an interactive, culturally adapted information-sharing process [11, 19] to address end-of-life and palliative care (EOLPC) for managing HF at home. This EOLPC intervention included guided practice of HF home care skills and engaging families in EOL care preference discussions. This model guides conversations about EOL advance directives and disease-specific HF palliative home caregiving [8, 9]. The model also addresses family caregiver HF home care burden [27]. Specifically, the FamPALcare nurse clinician used a coaching approach to prepare patients and caregivers with practical skills for specific HF palliative and end-of-life care. In addition, our FamPALcare clinician ensured that the intervention was culturally sensitive for rural Appalachian families [28]. The FamPALcare clinician and data collectors attended training sessions on culturally sensitive, coaching communication techniques for following the research protocols [29].

### Specific aims, directional hypotheses, and research questions

The following specific aims, directional hypotheses, and research questions were tested:

#### Specific Aim 1

To test whether the FamPALcare nursing care intervention could reduce the burden of family caregivers by preventing caregiver depression/anxiety and improve patient HF-related health status and depression/anxiety. The hypotheses and research questions are as follows.

*Aim 1.1* Compared to those in the control group at 3 and 6 months, the *caregivers* who received FamPALcare will have significant improvements in home caregiving burden and depression/anxiety scores.

*Aim 1.2* Compared to those in the control group at 3 and 6 months, *patients* in the FamPALcare group will experience greater improvement in HF-related health and depression/anxiety scores.

#### Specific Aim 2

To assess the feasibility and fidelity of the FamPALcare intervention.

*Aim 2.1.* What were the participant recruitment, enrollment, and retention outcomes?

*Aim 2.2.* How helpful was the FamPALcare as rated by participants on the anonymous 11-item Helpfulness Questionnaire?

*Aim 2.3.* What was the FamPALcare implementation cost using traditional tabulated cost-minimization analysis?

## Methods

### Design

This study used a randomized controlled trial (RCT) design to test the FamPALcare intervention which provides family caregivers with practical skills in caring for patients with HF as their status deteriorates. This deterioration increases palliative care needs as well as home caregiving burden [11]. The intervention group received routine standard care plus the FamPALcare intervention. The control group received standard care. Both groups completed the same measures on the same schedule. (See FamPALcare details in Intervention Section).

This RCT used random assignment to group with stratification of the patient gender to equalize distribution. SPSS version 29 was used to generate random numbers within each group. Each family dyad was randomly assigned to either the control or intervention group in a 1:1 fashion [11]. The researchers were blinded to the group assignments until informed consent was obtained. The Institutional Review Board of the University Health Sciences Center (WVU IRB 1709754988) approved the study protocol. The details of the study procedures have been described previously [11]. The RCT design was consistent with the National Template for Intervention Description and Replication (TIDieR) checklist and guide [30].

#### Sample and setting

Patients in this study were diagnosed with advanced HF (NYHA III/IV or Stages C/D) [2, 10] adjudicated by a cardiologist. Patients who had a heart transplant or left ventricular assist devices and those with other terminal illnesses (i.e., cancer) or severe dementia (i.e., Alzheimer’s disease) were excluded. The caregivers were named by the patients as the primary nonpaid persons who provided HF home care assistance. The caregivers were not all spouses; some were parents, daughters/sons, or other relatives. Caregivers with a disability who were unable to use FamPALcare intervention materials (i.e., Alzheimer’s disease) were excluded from the study. Both patients and caregivers signed informed consent forms to participate and were randomly assigned to group as a dyad. All participants were alert and able to read and write in English.

Our statistician calculated the sample size using alpha of 0.05, power = 1-β of 0.80, the conventional and most frequently selected in RCTs [31, 32] and used for other HF palliative care trials [33]. After adding subjects for potential attrition this study sample size was 36 families (18 patients and 18 caregivers per each group). Considering our sample size needs, we were able to find the number of HF patients, hospitalized and discharged, meeting our study criteria, in the rural Appalachian counties where the study would be conducted.

This sample size calculation coincided with findings from our previous pre-posttest HF intervention study. That clinical data found improvement of one standard deviation, a moderate effect size, in HF patient dyspnea [27]. Specifically, that data showed a reduction from moderate to mild respiratory symptoms [34]. Our FamPALcare clinician instructed patients and their caregivers on these successful home care approaches to relieve dyspnea [35].

The implications of selecting an 80% power are the risks of missing significant findings, when, in fact, there was a significant mean difference. Using a higher power would require much larger sample sizes [31, 32]. This risk is also considered in the discussion.

**Fig. 1:**
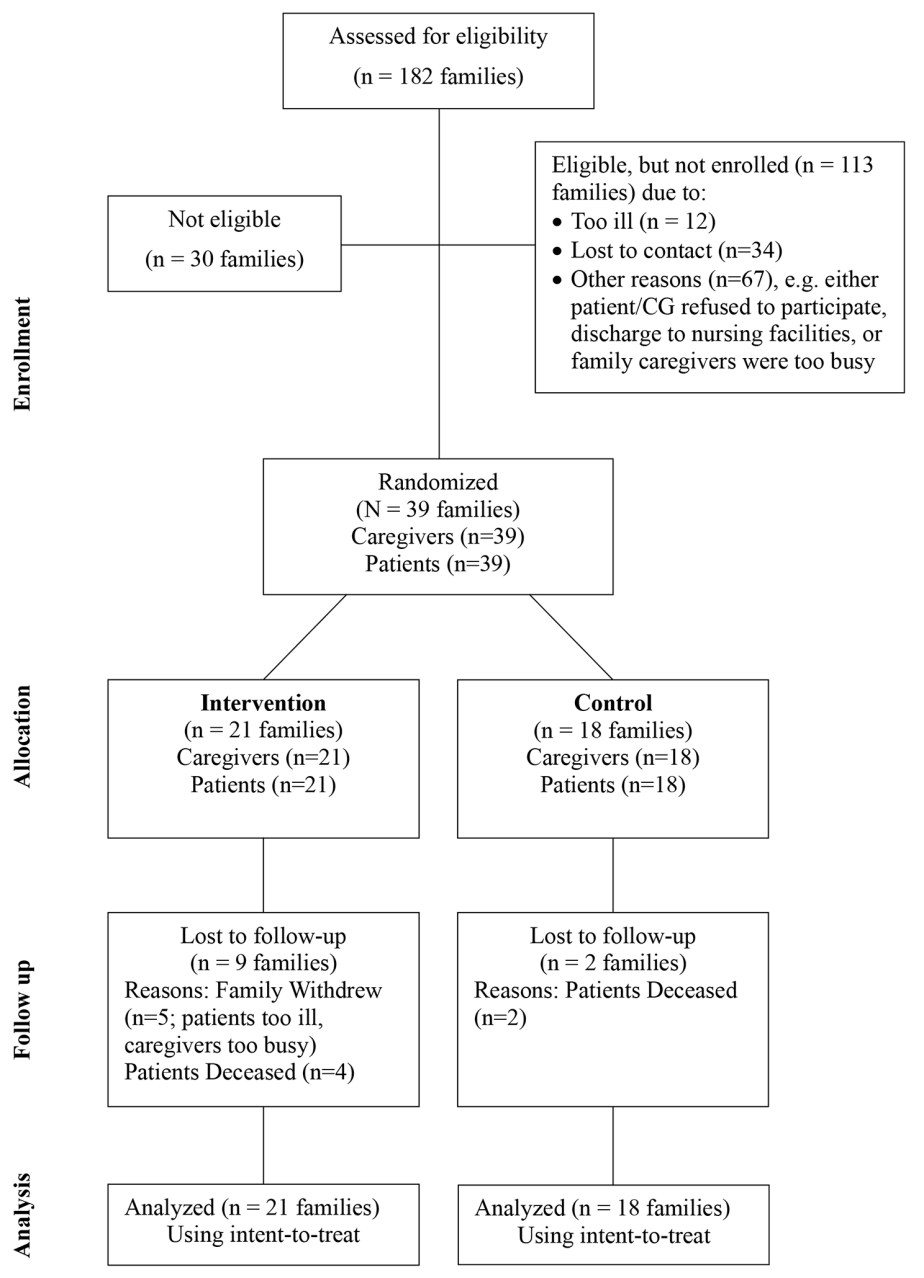
The Participants’ Recruitment and Enrollment Diagram (*N* = 39 families)

Patients were recruited from a large regional hospital in the WV, including HF inpatients (75%), outpatient units (23%), and self-referrals (2%). The cardiology nurse coordinator identified potential participants prospectively. Our trained research staff (nurse practitioners and registered nurses) contacted the eligible participants, explained the study, and obtained signed consent forms. All nurse recruiters had IRB permission to access patient records, which was consistent with the Health Insurance Portability and Accountability Act (HIPAA) regulations. Out of 152 eligible patients, 39 families (39 patients and 39 caregivers) agreed to participate and were randomly assigned to either the standard care or intervention group. See Fig. 1.

### Interventions

#### Standard care group

In this study, all patients and their family caregivers continued the standard health care prescribed by their healthcare providers. Patient standard HF care included routine educational materials given to them at the outpatient clinics or upon hospital discharge planning. Therefore, standard medical and nursing clinical care in both groups remained the same throughout the study. However, standard information was not specific to HF home palliative or EOL care topics.

#### Family home palliative care (FamPALcare) intervention group

Participants in the intervention group received standard care and the FamPALcare intervention. FamPALcare aims to provide family caregivers with practical skills for managing patient symptoms and deterioration of their HF status, addressing palliative care needs and home caregiving burden. The FamPALcare clinician nurse coached patients and their family caregivers on HF palliative symptom management and guided advanced directives discussions.

Participants in the intervention group received the materials manual for following along during the five telephone coaching sessions and the telephone follow-up calls used to reinforce FamPALcare HF home care and supportive palliative and end-of-life care discussions. Each coaching telephone session lasted from 60 to 90 min. The intervention sessions included the FamPALcare clinician developing rapport; assessing the families’ beliefs and concerns; addressing caregiver involvement in home care; and identifying each family’s HF home care needs. During the intervention sessions, the clinician played a crucial role in supporting home caregiving by reinforcing adherence to the patient’s prescribed low-sodium diet and medications, symptom monitoring, and timely reporting of deteriorating symptoms. The FamPALcare clinician taught the family methods to reduce HF symptoms (e.g., taking short relaxing naps during the day, using pillows to keep the head of the bed raised, undertaking small tasks one at a time, and using ways to avoid stress). The nurse also encouraged family caregivers to seek support from trusted individuals and faith-based groups, and to participate in local support programs if desired.

The FamPALcare manual provides families with step-by-step guides and visual illustrations for managing HF declines. These FamPALcare materials were designed to support home caregivers and patients with low literacy levels and to overcome barriers to providing adequate HF home care [19]. The FamPALcare intervention manual included pictures of an advanced scientific-based HF disease progression trajectory graph. The graph was used to explain the typical expected HF decline and how to handle common bothersome HF symptoms (e.g., breathlessness, anxiety/depression, edema, fatigue). In addition, examples of advanced directives form, Physician Orders for Scope of Treatment (POST) (e.g., do not resuscitate) forms, and local emergency contacts were provided [36]. The FamPALcare clinician recommended that forms be taken to the patient’s next healthcare provider’s appointment to sign and document in their medical records. Participants kept the FamPALcare manual with these informative and illustrated guidelines as well as contact lists for any available resources.

The manual materials were reviewed by older adults with advanced HF and their caregivers living in a rural WV county and evaluated as easy to follow and appropriate for HF home palliative and end-of-life care. The FamPALcare clinician followed the HF educational manual, which included coaching strategies for discussing the sensitive topic of end-of-life care [37] and included specific information regarding standard palliative and end-of-life care options. The FamPALcare clinician used an open-ended coaching conversation approach to facilitate patient wishes and preferences regarding end-of-life care.

To ensure that participants fully comprehended the information discussed during each FamPALcare session, the clinician utilized the effective “teach-back” technique [27]. By asking participants to describe what they had been taught, the nurse ensured that the information had been conveyed accurately and understood completely. The nurse also identified any topics that required further reinforcement for future retraining in the follow-up telephone calls. Furthermore, after three months, a follow-up phone call was made to provide additional support and encourage the continued practice of FamPALcare.

A second trained clinician who observed the FamPALcare clinician at randomly selected FamPALcare sessions, confirmed the fidelity of the intervention. This clinician documented the accuracy and consistency of the FamPALcare intervention implementation according to the manual [38]. Our research quality assurance measures included quarterly communication and intervention retraining and protocols for data collection monitoring [29].

### Data collection

Patients and caregivers completed the questionnaire surveys separately for privacy and to maintain data independence. All participants completed the surveys at baseline, 3 months, and 6 months. A trained researcher collected the data via telephone, while some of the participants were able to complete the questionnaires and mail them back to us. The data manager checked the completeness of the data, deidentified the surveys, and entered the data into the secured firewall-protected database. These research assistants were not involved in the intervention delivery. Secure data collection and storage were maintained per the national research policies.

### Measures

#### Caregiver and patient demographics

Both patients and their family caregivers provided their demographic information at baseline, including age, sex, race/ethnicity, level of education, employment, health insurance, years of HF diagnosis, and years of home HF caregiving. In measuring income, participants rated the adequacy of family income in relation to paying monthly expenses [39, 40].

#### Caregiver outcome measures

*Caregiving burden* was measured by the Zarit Caregiver Burden Interview (ZBI). The 12-item 5-point Likert scale was used to evaluate physical, social, financial, and emotional burdens of home caregiving [41]. Caregivers rated each item from never (score = 0) to nearly always (score = 4); possible total scores ranged from 0 to 48, in which a higher score indicated greater burden. A sample item was “Has your health suffered because of your involvement with your relative?”. In this study, Cronbach’s α = 0.92.

*Depressive/anxiety symptoms* were measured by a four-item Patient Health Questionnaire (PHQ-4) [42]. Family caregivers completed the PHQ-4. The response options varied from not at all (score = 0) to nearly every day (score = 3). Two items measure depression, and another 2 items measure anxiety. A total score *≥* 3 indicated depression/anxiety symptoms. Sample items are as follows: In the last 2 weeks, how often have you been bothered by “feeling nervous, anxious, or on edge?” “feeling down, depressed, or hopeless”? In this study, Cronbach’s α = 0.91 for caregiver samples.

*Helpfulness Rating Scale.* Upon completion of the FamPALcare, participants in the intervention group anonymously completed the 11-item helpfulness rating scale [27]. The response options were rated on a 5-point Likert scale ranging from strongly disagree/not helpful (score = 1) to strongly agree/very helpful (score = 5), in which a higher score indicated greater helpfulness of the intervention. One sample item was “The nurse showed me strategies to manage the symptoms of advanced heart failure at home (how to manage breathlessness, fatigue, depression, etc.)” and “It was helpful having a nurse (trained health care staff) provide step-by-step guidance and information on care options for advanced HF.” “Overall, I feel comfortable discussing my care options and wishes with my family and healthcare provider.” In this study, the helpfulness scale had a Cronbach’s α = 0.87.

#### Patient outcome measures

*Patient HF-related health status* was measured by the Kansas City Cardiomyopathy Questionnaire (KCCQ-12) [43]. The 12-item 5-point Likert scale was used to evaluate overall patient health status, including physical limitations, symptom frequency and severity, quality of life and social limitations. The standardized score scale ranged from 0 to 100. The norm-based score among HF patients was 50 (SD = 10). A higher score indicated better health status. In this study, Cronbach’s α = 0.73.

*Depressive/anxiety symptoms* were measured by a four-item Patient Health Questionnaire (PHQ-4) [42]. Patients completed the same PHQ-4 questionnaire (described under caregiver measures). In this study, Cronbach’s α = 0.91 for the patient samples.

### Data analysis

Descriptive statistics (frequencies, means, standard deviations) were used to summarize the caregiver and patient characteristics and program implementation costs. A generalized linear model (GLM) was used to test for significant differences between the control and intervention groups in terms of health outcome scores measured at baseline and at 3 and 6 months, controlling for covariate effects. The GLM is the extended version of the general linear model applicable to variables that are not normally distributed and is commonly used in small sample studies [44]. The GLM also provides a post hoc analysis that yields results similar to those of t tests. A strength of GLM is that it also provides repeated data collection time factors in post hoc analyses. Based on the conceptual framework and literature review, one-tailed group comparisons were used to test our a priori directional hypotheses using the Bonferroni adjustment. [45]. Thus, this post hoc paired comparison between groups can reveal significant differences between outcomes at baseline vs. 3 months and at baseline vs. 6 months. The intent-to-treat statistical approach was used, as all patients were included in the analysis with imputation means replacement [46]. Data analyses were conducted using SPSS (version 29).

This study used a traditional cost accounting method to calculate the cost of implementing FamPALcare. All expenses related to delivering the program were tabulated. The cost of the clinician delivering the intervention was calculated from an average of a nurse’s hourly salary ($50/hr). The costs of educational materials given to participants in the manual were tallied and summarized. A recruitment and enrollment diagram was used. (See Fig. 1.)

## Results

### Demographics

The average age of the family caregivers (*N* = 39) in this sample was 62.05 (SD = 13.14, range 36–86) years, and the average duration of caregiving was 9.45 (SD = 12.19) years. The majority of caregivers were females (76.90%) and White (87.20%). Approximately half of the family caregivers (48.70%) had completed a high school education or less, and 69.20% were married. Only 25.60% were employed. Most caregivers (95%) had health insurance coverage (e.g., Medicare, Medicaid, or other insurance). Notably, 25.60% reported that they could not make ends meet, while another 23.10% reported that they had just enough monthly income to pay their bills. See Table 1.

**Table 1:** Caregiver demographic characteristics and groups at baseline (*n* = 39)

Characteristics	All participants *N* = 39 (%)	Control group *n* = 18 (%)	Intervention group *n* = 21 (%)
**Age** (years), mean (SD) (range)	62.05 (13.14) (36–86)	59.55 (14.57) (36–86)	64.19 (11.73) (37–82)
**Length of caregiving (year)**, mean (SD), (range)	9.45 (12.19) (0–47)	8.41 (7.73) (1–28)	10.29 (15.01) (0–47)
**Sex**			
Male	9 (23.10)	4 (22.20)	5 (23.80)
Female	30 (76.90)	14 (77.80)	16 (76.20)
**Ethnicity**			
Non-Hispanic	38 (97.60)	17 (94.40)	21 (100.00)
Hispanic/Latino	1 (2.60)	1 (5.60)	0 (0.00)
**Race**			
White	34 (87.20)	15 (83.30)	19 (90.50)
Black/African American	4 (10.30)	2 (11.10)	2 (9.50)
American Indian/Alaska Native	1 (2.60)	1 (5.60)	0 (0.00)
**Education**			
High school or lower	19 (48.70)	11 (61.10)	8 (38.10)
Vocational/community college	5 (12.80)	2 (11.10)	3 (14.30)
Some college	8 (20.50)	2 (11.10)	6 (28.60)
Completed college or more	7 (17.90)	3 (16.70)	4 (19.00)
**Marital status**			
Married	27 (69.20)	14 (77.80)	13 (61.90)
Widowed/Divorced/Separated	8 (20.50)	2 (11.20)	6 (28.50)
Never married	4 (10.30)	2 (11.10)	2 (9.50)
**Employment**			
Employed	10 (25.60)	6 (33.30)	4 (19.00)
Unemployed	29 (74.40)	12 (36.70)	17 (81.00)
**Insurance**			
Medicare	17 (43.60)	6 (33.30)	11 (52.40)
Medicaid	10 (25.60)	6 (33.30)	4 (19.00)
Private/Other	10 (25.60)	4 (22.20)	6 (28.60)
No insurance	2 (5.10)	2 (11.10)	0 (0.00)
**Income adequacy**			
I can’t make ends meet	10 (25.60)	7 (38.90)	3 (14.30)
I have just enough; no more	9 (23.10)	4 (22.20)	5 (23.80)
I have enough, with a little extra	16 (41.00)	5 (27.80)	11 (52.40)
I always have money left over	4 (10.30)	2 (11.10)	2 (9.50)

The average age of the patients in this sample was 65.6 (SD = 13.72, range 32–88) years, and the average length of HF diagnosis was 6.53 (SD = 7.89) years. The majority of patients (66.70%) were male and white (87.20%), 56.40% had completed a high school education or less, and 62% were married. Most patients were unemployed or retired (87.17%), and 94.90% had health insurance coverage. Notably, 28% of patients reported that they could not make ends meet, while 23% reported having just enough income. The random assignment gender stratification helped equalize the male and female patients, but a slight disproportion between male/female was due to three families who were added to the intervention group due to drops out. See Table 2.

**Table 2:** Patient demographic characteristics and groups at baseline (*n* = 39)

Characteristics	All participants *N* = 39 (%)	Control group *n* = 18 (%)	Intervention group *n* = 21 (%)
**Age** (years), mean (SD) (range)	65.66 (13.72) (32–88)	65.77 (14.51) (32–88)	65.57 (13.38) (40–85)
**HF diagnosis** (years), mean (SD) (range)	6.53 (7.89) (< 1Yr-32)	6.83 (7.18) (1–29)	6.26 (8.65) (< 1Yr-32)
**Gender**			
Male	26 (66.70)	11 (61.10)	15 (71.40)
Female	13 (33.30)	7 (38.90)	6 (28.60)
**Ethnicity**			
Non-Hispanic	39 (100.00)	18 (100.00)	21 (100.00)
Hispanic/Latino	0 (0.00)	0 (0.00)	0 (0.00)
**Race**			
White	34 (87.20)	15 (83.30)	19 (90.50)
Black/African American	3 (7.70)	2 (11.10)	1 (4.80)
American Indian/Alaska Native	2 (5.10)	1 (5.60)	1 (4.80)
**Education**			
High school or lower	22 (56.40)	13 (72.20)	9 (42.80)
Vocational/community college	5 (12.80)	2 (11.10)	3 (14.30)
Some college	8 (20.50)	2 (11.10)	6 (28.60)
Completed college or more	4 (10.30)	1 (5.60)	3 (14.30)
**Marital status**			
Married	24 (61.50)	11 (61.10)	13 (61.90)
Widowed/Divorced/Separated	10 (25.70)	5 (27.80)	5 (23.80)
Never married	5 (12.80)	2 (11.10)	3 (14.30)
**Employment**			
Employed	5 (12.82)	2 (11.10)	3 (14.30)
Unemployed	34 (87.17)	16 (88.90)	18 (85.70)
**Insurance**			
Medicare	20 (51.30)	9 (50.00)	11 (52.40)
Medicaid	5 (12.80)	3 (16.70)	2 (9.50)
Private/Other (Military)	12 (30.80)	4 (22.20)	8 (38.60)
No insurance	2 (5.10)	2 (11.10)	-
**Income adequacy**			
I can’t make ends meet	11 (28.20)	8 (44.40)	3 (14.30)
I have just enough; no more	9 (23.10)	4 (22.20)	5 (23.80)
I have enough, with a little extra	13 (33.30)	5 (27.80)	8 (38.10)
I always have money left over	6 (15.40)	1 (5.60)	5 (23.80)

### Outcomes

#### Specific Aim 1.1

After controlling for caregiver age and years of HF caregiving, the measure GLM showed no significant difference between the control and intervention groups in terms of health outcome scores measured at baseline and at 3 and 6 months. But the GLM analysis showed that, at 3 months, compared to those in the control group, the caregivers who received FamPALcare had lower home care burdens (ZBI-12), t = 2.06, *p* = .025, and lower depression/anxiety (PHQ-4), t = 2.67, *p* = .007. However, there was no statistically significant difference in caregiver outcome measures (caregiving burden or depression/anxiety) at the 6-month end of the study. See Table 3.

**Table 3:** Comparison of caregiver data by group at baseline, 3 months, and 6 months (*N* = 39) from a fitted generalized linear model (GLM) model

Variables	Baseline			3-Months			6-Months		
	Control (*n* = 18)	Intervention (*n* = 21)	t (*p*)^3^	Control (*n* = 18)	Intervention (*n* = 21)	t (*p*)^3^	Control (*n* = 18)	Intervention (*n* = 21)	t (*p*)^3^
Zerit Burden^1^	13.56 (9.76)	9.57 (7.75)	1.42 (0.08)	13.38 (7.02)	9.48 (4.23)	2.06 (0.025)*	9.93 (7.16)	8.12 (3.67)	0.97 (0.17)
PHQ-4 scale^2^	4.33 (3.60)	3.52 (3.72)	0.69 (0.25)	4.53 (3.64)	2.06 (1.64)	2.67 (0.007)**	4.32 (3.80)	2.69 (2.01)	1.64 (0.06)

^1^ Zarit Burden measures caregiving burden

^2^ The PHQ-4 measures depression/anxiety

^3^ Statistical analysis used to compare scores between groups for each time point, t (p), *p < .05; **p < .01

#### Specific Aim 1.2

After controlling for patient age and years since the HF diagnosis, the repeated measures GLM showed no significant difference between the control and intervention groups in terms of health outcome scores measured at baseline and at 3 and 6 months. The GLM analysis showed that there was no significant difference in patient outcome measures (health status, depression/anxiety) at baseline or at the 3-month follow-up. However, compared to those in the control group at 6 months, the patients in the FamPALcare group had higher HF-related health status (KCCQ) scores (t = -1.89, *p* = .03) and lower depression/anxiety (PHQ-4) scores (t = 2.06, *p* = .02). See Table 4.

**Table 4:** Comparison of patient data at baseline, 3 months, and 6 months (*N* = 39) according to the fitted generalized linear model (GLM) model

	Baseline			3-month			6-month		
**Variables**	**Control** **(n = 18)**	**Intervention** **(n = 21)**	**t (p)** ^3^	**Control** **(n = 18)**	**Intervention** **(n = 21)**	**t (p)** ^3^	**Control** **(n = 18)**	**Intervention** **(n = 21)**	**t (p)** ^3^
KCCQ-12^1^	27.11 **(**14.63)	26.79 (18.30)	0.06 (0.48)	44.57 (24.54)	54.73 (22.58)	-1.35 (0.19)	46.00 (23.08)	59.49 (21.29)	-1.89 (0.033)*
PHQ-4^2^	5.89 (3.67)	5.71 (4.75)	0.13 (0.45)	3.90 (2.86)	2.97 (2.46)	1.09 (0.14)	4.10 (3.08)	2.44 (1.89)	2.06 (0.02)*

^1^ KQQC-12 measures patients’ HF-related health status

^2^ The PHQ-4 measures depression/anxiety

^3^ Statistical analysis used to compare scores between groups for each time point, t (p), *p < .05; **p < .01

#### Specific Aim 2.1

##### Recruitment, enrollment, and retention

A total of 182 patients’ electronic medical records were screened, with IRB approval, by two trained research assistants (registered nurses). Of the 182 patients screened, 152 were eligible. Of the 152 individuals who were eligible, 39 families (patients/family caregiver dyads) agreed, signed consent to participate, and were randomized to control (*n* = 18 dyads) or intervention (*n* = 21 dyads) group. There were 113 patients who were eligible but did not enroll in the study. The reasons for nonparticipation were that the patients were too ill (*n* = 12), had lost contact after hospital discharge (*n* = 34), or had other reasons (*n* = 67); e.g., the patients were discharged to nursing facilities or family caregivers were too busy to enroll. See Fig. 1. There was a 28.2% attrition rate (five family dyads withdrew, and six patients died). Overall, the feasibility of this FamPALcare study was confirmed by successful completion of all dyad session implementations, complete data collection, and continuing rural residents’ support for HF FamPALcare.

#### Specific Aim 2.2

At the 6-month follow-up, participants completed the anonymous 11-item Likert-type intervention helpfulness rating scale. The average score across 11 items was M = 4.46 out of 5 (SD = 0.49). The means of the items ranged from 3.50 to 5.00, indicating the helpfulness of the FamPALcare intervention and materials, which were all rated highly above the midpoint. See Table 5.

**Table 5:** FamPALcare Helpfulness ratings by participants at 6-months

Rate how much you agree with the following from 1 to 5, strongly disagree/not helpful (score = 1) and strongly agree/very helpful (score = 5)	Mean (SD)
1. Seeing the illness trajectory graph, I can anticipate common symptoms in advanced heart failure (i.e., breathlessness, depression, anxiety, fatigue).	4.33 (0.52)
2. The nurse showed me strategies to manage the symptoms of advanced heart failure at home (how to manage breathlessness, fatigue, depression, etc.).	4.83 (0.41)
3. It is important to learn about care choices about advance heart failure for comfort and supportive care when my symptoms become worse (e.g. Hospice Care, Home Health Care, or Home Hospice Care, etc.).	4.67 (0.52)
4. It is important to bring an Advance Directive and related forms to the clinic appointment to discuss with health care providers.	4.67 (0.52)
5. Documenting my advance heart failure care options in the Advance Directive form will assure that my wishes will be honored.	4.67 (0.52)
6. The discussion about available resources and/or home assistance in my community to support home care for my family was useful.	4.50 (0.84)
7. It was helpful having a nurse (trained health care staff) provide step-by-step guidance and information on care options for advance heart failure.	4.67 (0.52)
8. The explanation of the Advance Directive, POST (pink form including a do-not-resuscitate order), Living Will, and/or Durable Power of Attorney for Healthcare was helpful.	4.83 (0.41)
9. The nurse gave me time to ask questions about care options.	4.67 (0.52)
10. I was comfortable during the discussion with the nurse.	4.67 (0.52)
11. Overall, I feel comfortable discussing my care options and wishes with my family and healthcare provider.	4.83 (0.41)

#### Specific Aim 2.3

The costs of personnel included (a) the FamPALcare clinician time for week 1 = $50 of average nurse salary per hour × 2 h = *$100*; (b) plus 4 subsequent weeks $50 × 4 × 0.75 h = *$150*; (c) 30-minute reinforcement at 6-month $50 × 0.5 h = *$25;* and (d) the training time for the observation for two clinicians × 2 h = $50 × 4 h = $200 divided by the number of intervention families (*n* = 21), average cost = *$10*/family). The educational material cost was $25, which included pamphlets, booklets, sheet protectors, flexible binders, and other office supplies. The mailing and postal costs were $6.50 per family. Thus, the total implementation cost for one family completing all the sessions was $316.50 ($285 + $25 + $6.50). This cost analysis excluded participants’ small honorarium, research personnel program development time, and research questionnaire duplication costs. The grant covered the cost. Future studies will also calculate projected administrative costs for sustaining FamPALcare.

## Discussion

The results of this study are based on the outcome measures from the Aim 1 hypotheses. Patients in the intervention group had significantly greater scores for HF-related health status (*p* < .05) and lower depression/anxiety scores at 6 months, compared to baseline. The family caregivers in the intervention group had significantly lower scores on caregiving burden (*p* < .05) and depression/anxiety (*p* < .01) at 3 months, compared to baseline. The unique delivery of this FamPALcare intervention that led to these positive results included the coaching strategies used to provide practical home care skills. These strategies engaged family caregivers and patients in discussing the sensitive topic of end-of-life care and specific HF symptom management information [47]. Further, our FamPALcare clinician has EOLPC certificates and extensive experiences in home palliative care. These expert sensitive approaches are essential for palliative and end-of-life care [35].

The patient improvements occurring at 6 months but not at 3 months may have been related to their poor baseline HF health status and serious depression/anxiety. Patient HF status, likely improved related to symptom control technique gained from FamPALcare. Patients may need a longer period of time to practice controlling HF symptoms at home and to discuss their advanced directives. The caregiver improvements occurring at 3 months but not at 6 months. Possibly the lack of a significant difference in caregivers at 6 months was related to the timing of the telephone intervention sessions in the FamPALcare. The telephone reinforcement intervention session was closer to the 3-month follow-up than to the 6-month follow-up. As a result of these findings, we recommended future studies delivered the intervention across 6 months, followed by monthly reinforcement calls for 6 months. Further, future studies need larger sample sizes. In addition, based on the systematic reviews [48] and the few HF palliative care RCTs [22, 49, 50], most of the intervention lasted for 3 to 6 months. Across the 15 studies reviewed, follow ups lasting beyond 3 months reported positive improvement on patient symptom burden but varied caregiving outcomes [25], possibly due to varied home caregiving responsibilities as patients’ health decline after 6 months. The monthly follow-up for one year allows the clinician to provide families new information as patients decline. These methods could strengthen the FamPALcare intervention, which was evaluated highly and can easily be delivered by telephone.

As shown in other chronic disease studies, caregivers experiencing high levels of burden (negative physical and mental impact) become ill themselves [5, 6]. Therefore, caregivers should be enrolled in studies to reduce their burdens. Improved caregiving burden and depression/anxiety is critical for HF home care adjustment. Our RCT showed an improvement in both caregiving burden and depression/anxiety. This mental health finding was also present in other HF palliative care clinical trials [22, 49]. Those trials used similar measures, although there were very few studies involving family caregivers [25, 50] or providing advanced directives [51]. Additionally, participants with HF in these other palliative care RCTs often had to travel to meet with interventionists [49]. The FamPALcare remote contact allows for caregiver involvement and no travel, making it more practical for participants.

Following hospital discharge, almost one-quarter of patients hospitalized with severe HF expressed a preference to not be resuscitated. Yet, a substantial proportion of those patients changed their minds to be resuscitated due to the common HF improvement within two months of discharge [52]. Thus, early repeated and long-term palliative care discussions are warranted regarding sporadic HF decline then improvements, from medication or internal cardiac device changes, and desired continuation of resuscitation [8, 53]. Reports indicate that early EOLPC can help address patient goals and family preferences regarding treatment [54].

A recent review of HF EOL care found that major needs of patients and their caregivers are relate to communication. [55]. Indeed, as we observed and were informed of by participants that communication with health professionals and families often lack clarity, and information about HF and instruction for home care was inadequate [23, 56]. Such family education can be satisfied by key aspects of our FamPALcare, particularly the clinician care coaching communication and HF management. Our findings can support the design of new strategies including establishing rapport using clinicians from the same communities and including providing home care training specific to HF care. The FamPALcare clinician developed a rapport with participants which enhanced their engagement in the intervention sessions. Our FamPALcare clinician was born and raised, obtained her nursing education, and lives in Appalachia. This familiarity supports trust, study recruitment, and culturally sensitive approaches with families [19, 57, 58].

Based on a recent Genworth survey [59], the median cost of in-home care services in the state of West Virginia (homemaker services or home health aide) was $4,767/month, $5,500/month for assisted living, and $11,619/month for semiprivate rooms in a nursing home. The implementation cost of FamPALcare was $316.50 per family, considerably less than that of in-home care, an assisted living or nursing facility, a single emergency department visit, or one inpatient hospitalization for HF.

A Cochrane Systematic Review reported costs of palliative home care [60], and as in this study, supported low cost telephone/mail delivery to rural families. Given the shortage of palliative care providers in rural areas, studies using low-cost novel telehealth interventions are essential [61]. FamPALcare could benefit HF patients and their family caregivers throughout the course of the disease. It is commonly reported that patient deaths occur within 5 years after the initial HF diagnosis [62], so early EOL preparation is needed.

Participants gave the intervention high ratings on the anonymous 11-item Helpfulness subscale of the FamPALcare questionnaire. (Table 5) Methods to reduce caregiving burden and family support were also lauded. Evidence of the value of remote monitoring was demonstrated by the high overall FamPALcare participant rating scores (M = 4.47 out of 5). Participants reported “being comfortable during the discussion with the nurse clinician, even when talking about HF care options and EOL wishes.” No specific measurements after each FamPALcare telephone call session were posed.

The impact of FamPALcare was highly rated as excellent in guiding management of HF home care and planning advance directives. Caregivers were satisfied and reported the benefits of the information on how to manage the symptoms of advanced HF at home (i.e., breathlessness, fatigue, edema, depression). Caregivers also rated they were comfortable discussing EOLPC options and preferences with their extended family members and healthcare providers. Unfortunately, caregivers and patients were unaware of the limited resources in their rural areas. Linking them to available community resources, could ease the overwhelming burden of living with HF and caring for loved ones at home in rural locations. Further, telephone follow-up is recommended within 30 days after hospital discharge and every 3 months to assess patient and caregiver needs.

### Study challenges and resolutions

There was an expected delay in our initial enrollment during winter (December to March) due to extreme weather and road conditions in rural Appalachia and COVID-19 restrictions (December 2019 through Mid-2022). However, only slight variation in participant intervention timelines was necessary in a few cases. The flexibility of the intervention schedule helped with the retention of participants in the study. Thirteen of the families enrolled in the study indicated that COVID-19 had impacted them, and 30% of them did not obtain sufficient healthcare during that time [63]. The families reported difficulty obtaining medications and rescheduling appointments. Such appointments and treatment delays may have impacted their home HF care management and increased the burden of home caregiving for family caregivers. This, too may have impacted the 6-month caregiver results.

Participant enrollment was completed as planned, but there was a higher attrition rate than anticipated. Of the 39 families enrolled, 11 could not complete the 6-month endpoint (11 out of 39 = 28.2% attrition). This attrition was due to five families withdrawing because patients became too ill or caregivers too busy, and six patients died. All procedures outlined in the retention strategies were successful in prior longitudinal clinical trial studies [64]. However, retention in this study was impacted by weather, rural isolation, the severity of HF disease among patients, and overwhelmed caregivers. This study’s follow-up reinforcement telephone calls and our previous research indicated that family caregivers need HF-specific repeated communication, home care clarification, and reassurance to prepare them for sustaining HF home EOL and palliative care [65].

### Implications

Telephone contact for FamPALcare visits promptly after hospital or ER discharge could be helpful for these families [66]. Preparing caregivers for home care has been shown to reduce older adult hospitalizations [67] and hospice referrals, improve their pain control [68], and their economic burden [69]. Implementing HF home care earlier is critical to prevent or delay HF decline. Patients with advanced HF and their family members benefit from early information on home palliative discussions and EOLPC care interventions [54, 55].

Home-based palliative care can meet the needs of people with a life-limiting illness (e.g., advanced HF) who are neither hospitalized nor hospice-eligible. Palliative care services and guidance can be delivered by clinicians in primary care and specialty care practices, as well as through home health agencies, home-based medical companies, hospices, and health systems [13]. In underserved rural areas, mailing materials and telephone connections can ensure that families are supplied with the materials needed to manage their disease. Our FamPALcare intervention provides methods that can effectively ease the multitude of burdens associated with HF disease for both patients and their family caregivers.

Palliative care and hospice care share common goals of symptom control, provide comfort, and support patients and their families during challenging times. Palliative care is available to a person at any stage of a serious or long-term illness, while hospice care is for a person with a terminal illness, determined by a doctor to have less than 6 months to live [12, 13]. This rural area has few hospice services. Most rural Appalachian areas have both limited palliative and hospice care services. Yet, Appalachian culture uses extensive family, neighbor, and religious resources that are routinely mobilized. These resources are listed in our FamPALcare manual to ensure patients and caregivers are aware of them.

### Limitations

Limitations of this study were greater than desired (28% vs. expected 20%) attrition. However, the repeated measures in this study strengthened this data results by increasing measurement independent variables over time. The study was conducted during the unprecedented COVID-19 pandemic, which impacted enrollment and the attrition rate. Another limitation is that there were a few nonwhite participants in the study, which aligns with the 93% of the non-Hispanic White population in WV [70]. Our findings may not be generalizable to nonwhite families managing HF. Further, considering the few available palliative care professionals, extending the FamPALcare intervention sessions to 6 months and continuing the follow-up calls through one year may be possible by telephone or internet contacts. Additionally, a larger sample size is recommended for future studies that include multiple variables.

## Conclusion

The feasibility, fidelity, and helpful nature of the FamPALcare palliative and end-of-life rural HF home care intervention were verified. The improved scores on patient HF health status and caregiver burden and lower scores on patient and caregiver depression/anxiety demonstrated the benefit of the FamPALcare intervention. The cost-effectiveness and positive interventional evaluation from the FamPALcare study’s HF patients and caregivers will guide the planning of subsequent clinical trials.

## Data Availability

Data is provided within the manuscript only.

## Abbreviations

HF: Heart failure
EOLPC: End-of-life palliative care
FamPALCare: Family palliative care intervention
IRB: Institutional Review Board
WV: West Virginia
SMC: Safety Monitoring Committee
AHA: American Heart Association
TIDieR: Template for intervention description and replication
